# An evaluation of the polymorphisms Ins16bp and Arg72Pro in *p53* as breast cancer risk modifiers in *BRCA1* and *BRCA2* mutation carriers

**DOI:** 10.1038/sj.bjc.6604624

**Published:** 2008-09-09

**Authors:** A Osorio, M Pollán, G Pita, R K Schmutzler, B Versmold, C Engel, A Meindl, N Arnold, S Preisler-Adams, D Niederacher, W Hofmann, D Gadzicki, A Jakubowska, U Hamann, J Lubinski, A Toloczko-Grabarek, C Cybulski, T Debniak, G Llort, D Yannoukakos, O Díez, B Peissel, P Peterlongo, P Radice, T Heikkinen, H Nevanlinna, P L Mai, J T Loud, L McGuffog, A C Antoniou, J Benitez

**Affiliations:** 1 Human Cancer Genetics Programme, Human Genetics Group, Spanish National Cancer Centre (CNIO), Madrid, Spain; 2 National Epidemiology Centre, Instituto de Salud Carlos III & CIBERESP, Madrid, Spain; 3 Human Cancer Genetics Programme, Genotyping Unit, Spanish National Cancer Centre (CNIO), Madrid, Spain; 4 Division of Molecular Gynaeco-Oncology, Department of Obstetrics and Gynaecology, University of Cologne, Cologne, Germany; 5 Institute for Medical Informatics, Statistics and Epidemiology, University of Leipzig, Leipzig, Germany; 6 Department of Obstetrics and Gynaecology, Technical University, Munich, Germany; 7 Department of Obstetrics and Gynaecology, University of Schleswig-Holstein, Campus Kiel, Germany; 8 Institute of Human Genetics, University of Münster, Münster, Germany; 9 Department of Obstetrics and Gynaecology, Molecular Genetics Laboratory, University of Düsseldorf, Düsseldorf, Germany; 10 Institute of Human Genetics, Charite-University Medical Centre, Berlin, Germany; 11 Institute of Cellular and Molecular Pathology, Medical University, Hannover, Germany; 12 Department of Genetics and Pathology, Pomeranian Medical University, Szczecin, Poland; 13 German Cancer Research Center, Molecular Genetics of Breast Cancer, Heidelberg, Germany; 14 Cancer Genetic Counselling Progam, Catalan Institute of Oncology (ICO), Barcelona, Spain; 15 Molecular Diagnostics Laboratory IRRP, NCSR Demokritos, Athens, Greece; 16 Genetics Service, Hospital de Sant Pau, Barcelona, Spain; 17 Oncogenetics Laboratory, Hospital Vall d'Hebron, Barcelona, Spain; 18 Medical Genetics Service, Fondazione IRCCS Istituto Nazionale dei Tumori, Milan, Italy; 19 Unit of Genetic Susceptibility to Cancer, Department of Experimental Oncology, Fondazione IRCCS Istituto Nazionale dei Tumori, Milan, Italy; 20 IFOM, Fondazione Istituto FIRC di Oncologia Molecolare, Milan, Italy; 21 Department of Obstetrics and Gynecology, Helsinki University Central Hospital (HUCH), Helsinki, Finland; 22 Clinical Genetics Branch, Division of Cancer Epidemiology and Genetics, National Cancer Institute, NIH, DHHS, Bethesda, MD, USA; 23 Cancer Research UK Genetic Epidemiology Unit, Department of Public Health and Primary Care, University of Cambridge, Cambridge, UK

**Keywords:** *BRCA1*, *BRCA2*, *p53*, breast cancer

## Abstract

The close functional relationship between p53 and the breast cancer susceptibility genes *BRCA1* and *BRCA2* has promoted the investigation of various polymorphisms in the *p53* gene as possible risk modifiers in BRCA1/2 mutation carriers. Specifically, two polymorphisms in *p53*, c.97-147ins16bp and p.Arg72Pro have been analysed as putative breast cancer susceptibility variants, and it has been recently reported that a *p53* haplotype combining the absence of the 16-bp insertion and the presence of proline at codon 72 (No Ins-72Pro) was associated with an earlier age at the onset of the first primary tumour in *BRCA2* mutation carriers in the Spanish population. In this study, we have evaluated this association in a series of 2932 BRCA1/2 mutation carriers from the Consortium of Investigators of Modifiers of *BRCA1* and *BRCA2*.

Given the involvement of *p53* in cell cycle control, DNA repair and apoptosis, the role of this gene in cancer susceptibility has been extensively studied. Specifically, two polymorphisms in *p53*, c.97-147ins16bp and p.Arg72Pro have been analysed as putative breast cancer susceptibility variants, although not all studies have yielded consistent results (Weston *et al*, 1997; Wang-Gohrke *et al*, 1998; Suspitsin *et al*, 2003; Damin *et al*, 2006; Baynes *et al*, 2007; Costa *et al*, 2008). The Arg72Pro single-nucleotide polymorphism (SNP) has gained special attention, as there is consistent evidence of functional differences in apoptotic rates between the Arg and Pro variants (Biros *et al*, 2002; Wu *et al*, 2002; Dumont *et al*, 2003). In addition, the close functional relationship between *p53* and the breast cancer susceptibility genes *BRCA1* and *BRCA*2 (Jonkers *et al*, 2001; Ongusaha *et al*, 2003; Liu *et al*, 2007) has promoted the investigation of the Arg72Pro SNP as a possible risk modifier in *BRCA1/2* mutation carriers (Martin *et al*, 2003). Indeed, it was recently reported that a *p53* haplotype combining the absence of the 16-bp insertion and the presence of proline at codon 72 (No Ins-72Pro) was associated with an earlier age at onset of the first primary tumour in *BRCA2* mutation carriers in the Spanish population (Osorio *et al*, 2006). In this study, we have evaluated this association in a series of 2932 *BRCA1/2* mutation carriers from the Consortium of Investigators of Modifiers of *BRCA1* and *BRCA2* (CIMBA) (Chenevix-Trench *et al*, 2007).

## Materials and Methods

### Patients

A total of 2088 *BRCA1* mutation carriers, 841 *BRCA2* mutation carriers and 3 carriers of mutations in both genes ascertained from eight centres participating in CIMBA were included in this study (Table 1). The inclusion criteria for subjects is described elsewhere (Chenevix-Trench *et al*, 2007).

### Genotyping

Genotypes for the two polymorphisms – Ins16bp and Arg72Pro – were determined for each sample using previously described methodology (Osorio *et al*, 2006). In some cases, the Ins16bp SNP was genotyped by DHPLC on the WAVE HT system (Transgenomic, Omaha, NE, USA) using an acetonitrile gradient and profiles analysed with the Navigator™ software (Transgenomic), and the Arg72Pro SNP was genotyped by Taqman (Applied Biosystems, Foster City, CA, USA). Hardy–Weinberg equilibrium (HWE) for each polymorphism was tested using the likelihood ratio test among unrelated individuals. The German Consortium of Hereditary Breast and Ovarian Cancer (GCHBOC) study gave HWE *P*-values of 0.005 and 0.043 for Ins16bp and Arg72Pro, respectively; therefore, all genotypes from that patient subset were confirmed by an alternative technique (DHPLC). In addition, individuals homozygous for Ins16bp were directly sequenced. The concordance rate was 100% in both instances; accordingly, the GCHBOC mutation carriers were included in all subsequent analyses. Call rates ranged between 92 and 100% across studies.

### Statistical analysis

Haplotypes were imputed using the R-package ‘hapassoc’ (Burkett and McNeney, 2006). Associations of individual haplotypes with time to breast cancer or ovarian cancer diagnosis were evaluated using weighted Cox proportional hazards models, using age as the time variable (Antoniou *et al*, 2005). Carriers were censored at the first occurrence of breast or ovarian cancer or bilateral prophylactic mastectomy. To allow for correlations between members of the same family, Huber and White robust estimators of variance were used, considering women clustered within families (Huber, 1967). The most frequent haplotype was taken as the reference and all other haplotypes were included in the multivariate model considering the number of copies of that particular variant. Models were adjusted for ethnicity, birth cohort and centre of recruitment. The analysis considered *BRCA1* and *BRCA2* mutation carriers separately (Table 2) and all carriers combined (data not shown).

## Results and Discussion

Genotype distributions and frequencies for the Ins16bp and Arg72Pro polymorphisms are shown in Table 1. Allele frequencies were similar to those previously published (Osorio *et al*, 2006), and genotype frequencies were consistent with HWE, except for the carriers from GCHBOC (see Materials and Methods). Haplotypes were inferred, and haplotype- and genotype-specific hazard ratios were estimated separately for each of breast (Table 2) and ovarian cancer (data not shown), among *BRCA1* and *BRCA2* mutation carriers. No evidence of association was found for any of the genotypes or haplotypes analysed with either breast or ovarian cancer risk, including the No Ins-72Pro haplotype, previously reported to be associated with an increased risk to develop a first primary tumour before 35 years of age in *BRCA2* mutation carriers (Osorio *et al*, 2006).

To confirm that this negative result was not due to the different analytic approach performed in this study, we carried out a logistic regression analysis, as was done in the original study (Osorio *et al*, 2006), considering those with age at diagnosis younger than 35 as cases, and did not find a positive association between early diagnosis and this haplotype. In the original study, the result was corroborated by a functional assay (Osorio *et al*, 2006), in which a decrease in apoptotic rate was found to be associated with the No Ins-72Pro haplotype. However, although concordant, both the genetic and the functional studies were limited by the small sample size (265 and 24 individuals, respectively), as reflected in the marginal statistically significant results described in that report.

In summary, the previously reported association of the No Ins-72Pro haplotype in *p53* with an increased cancer risk in *BRCA2* mutation carriers (Osorio *et al*, 2006) has not been validated in a larger series proceeding from the Consortium of Investigators of Modifiers of BRCA1 and BRCA2 (CIMBA). In this series of 2932 BRCA1/2 mutation carriers, no evidence of modification of breast or ovarian cancer risk by any of the two polymorphisms, Ins16bp and Arg72Pro, or their haplotype combinations has been detected. The lack of confirmation of a previously reported association found in a much smaller series highlights the necessity of international collaborative efforts aimed at achieving the statistical power required to reach reliable definitive conclusions in genetic association studies.

## Figures and Tables

**Table 1:** Genotype distribution of the two *p53* polymorphisms in the *BRCA1* and *BRCA2* mutation carriers by participating study

			**Ins16bp *N* (%)**				**Arg72Pro *N* (%)**			
**Study**	**Country of residence**	**Ascertainment basis**	**No Ins**	**No Ins/16bp Ins**	**16bpIns**	**Total**	**Arg72Arg**	**Arg72Pro**	**Pro72Pro**	**Total**
CNIO	Spain and Greece^a^	Clinic	335 (74.12%)	105 (23.23%)	12 (2.65%)	452	281 (56.31%)	176 (35.27%)	42 (8.42%)	499
MBCSG	Italy	Clinic	190 (65.07%)	91 (31.16%)	11 (3.77%)	292	156 (50.81%)	135 (43.97%)	16 (5.21%)	307
DKFZ	Germany, Pakistan, Colombia	Clinic	128 (74.42%)	41 (23.84%)	3 (1.74%)	172	87 (51.18%)	67 (39.41%)	16 (9.41%)	170
GCHBOC^b^	Germany	Clinic	593 (75.16%)	171 (21.67%)	25 (3.17%)	789	474 (56.97%)	294 (35.34%)	64 (7.69%)	832
HEBCS	Finland	Clinic	148 (78.72%)	39 (20.74%)	1 (0.53%)	188	96 (51.06%)	79 (42.02%)	13 (6.91%)	188
NCI	United States	Clinic	160 (73.06%)	56 (25.57%)	3 (1.37%)	219	96 (50.26%)	81 (42.41%)	14 (7.33%)	191
IHCC	Poland	Clinic	458 (67.25%)	202 (29.66%	21 (3.08%)	681	328 (48.16%)	289 (42.44%)	64 (9.40%)	681
Total			2012 (72.04%)	705 (25.24%)	76 (2.72%)	2793^c^	1518 (52.93%)	1121 (39.09%)	229 (7.98%)	2869

Abbreviations: GCHBOC=German Consortium of Hereditary Breast and Ovarian Cancer; HWE=Hardy–Weinberg equilibrium.

a The CNIO series consisted of samples from the Spanish Consortium for the Study of Genetic Modifiers of *BRCA1* and *BRCA2* and the NCSR Demokritos, Athens (Greece). Cases from the original study were included in the analysis (Osorio *et al*, 2006).

b Deviation from HWE with *P*-values of 0.005 and 0.043 was observed for Ins16bp and Arg72Pro, respectively.

c Missing genotypes are not included in the totals. Owing to technical difficulties, more failed genotypes were observed for the Ins16bp polymorphism.

**Table 2:** Haplotype frequencies^a^ by mutation and disease status and HR estimates for breast cancer

	**Unaffected (%)**	**Affected (%)**	**HR**	**95% CI**	***P*-value**
BRCA1 *mutation carriers*					
*p53* haplotype					
No Ins-Arg72/No Ins Arg72^b^	49.60	50.50	1.00		
**No Ins-72Pro**					
**One**^c^	**23.30**	**23.30**	**1.05**	**0.84–1.32**	**0.64**
**Two**^d^	**2.80**	**2**	**0.80**	**0.47–1.38**	**0.42**
Ins16bp-72Pro					
One	23.80	23.10	1.03	0.83–1.28	0.79
Two	2.20	2.10	1.16	0.54–2.50	0.70
Ins16bp-Arg72					
One	4	3.30	1.42	0.96–2.10	0.08
Two	—	—	—	—	—
					
*BRCA2 mutation carriers*					
*p53* haplotype					
No Ins-Arg72/No Ins Arg72	47.50	55.90	1.00		
**No Ins-72Pro**					
**One**	**26.50**	**23.60**	**0.82**	**0.53–1.26**	**0.35**
**Two**	**0.90**	**0.90**	**1.41**	**0.56–3.55**	**0.46**
Ins16bp-72Pro					
One	26.50	19.40	0.81	0.52–1.27	0.36
Two	2.10	2.20	0.72	0.14–3.86	0.70
Ins16bp-Arg72					
One	2	2.70	1.11	0.42–2.97	0.83
Two	—	—	—		

Abbreviations: CI=confidence interval; HR=hazard ratio.

HRs corresponding to the haplotype associated with increased cancer risk in the original study are in bold.

a Haplotypes were established or inferred only in those cases who had data for both polymorphisms.

b Those individuals who were homozygous for the haplotype containing the common allele for both polymorphisms were considered as the reference group.

c Individuals harbouring at least one given haplotype (heterozygous or homozygous)

d Individuals homozygous for a given haplotype.
